# Activation of Defense Mechanisms against Pathogens in Mosses and Flowering Plants

**DOI:** 10.3390/ijms14023178

**Published:** 2013-02-04

**Authors:** Inés Ponce de León, Marcos Montesano

**Affiliations:** 1 Departamento de Biología Molecular, Instituto de Investigaciones Biológicas Clemente Estable, Avenida Italia 3318, CP 11600, Montevideo, Uruguay; 2 Laboratorio de Fisiología Vegetal, Centro de Investigaciones Nucleares, Facultad de Ciencias, Mataojo 2055, CP 11400, Montevideo, Uruguay; E-Mail: montesano@cin.edu.uy

**Keywords:** *Physcomitrella patens*, flowering plants, defense mechanisms, ROS, cell wall, programmed cell death, defense genes, defense hormones

## Abstract

During evolution, plants have developed mechanisms to cope with and adapt to different types of stress, including microbial infection. Once the stress is sensed, signaling pathways are activated, leading to the induced expression of genes with different roles in defense. Mosses (Bryophytes) are non-vascular plants that diverged from flowering plants more than 450 million years ago, allowing comparative studies of the evolution of defense-related genes and defensive metabolites produced after microbial infection. The ancestral position among land plants, the sequenced genome and the feasibility of generating targeted knock-out mutants by homologous recombination has made the moss *Physcomitrella patens* an attractive model to perform functional studies of plant genes involved in stress responses. This paper reviews the current knowledge of inducible defense mechanisms in *P. patens* and compares them to those activated in flowering plants after pathogen assault, including the reinforcement of the cell wall, ROS production, programmed cell death, activation of defense genes and synthesis of secondary metabolites and defense hormones. The knowledge generated in *P. patens* together with comparative studies in flowering plants will help to identify key components in plant defense responses and to design novel strategies to enhance resistance to biotic stress.

## 1. Introduction

Plants are in permanent contact with a variety of microbial pathogens, such as fungi, oomycetes, bacteria and viruses. To ward off these pathogens, plants must recognize the invaders and activate fast and effective defense mechanisms that arrest the pathogen. Perception of the pathogens is central to the activation of a successful plant defense response. Plant cells are capable of sensing evolutionarily conserved microbial molecular signatures, collectively named pathogen-associated molecular patterns (PAMPs) or microbe-associated molecular patterns (MAMPs), by plant pattern recognition receptors (PRRs) [1–3]. MAMPs are molecules that are essential for microbe fitness and survival and are conserved between different species, resulting in an efficient form to sense the presence of pathogens by the plant. Perception of PAMPs by PRRs activates an immune response, referred to as PAMP-triggered immunity (PTI), which provides protection against non-host pathogens and limits disease caused by virulent pathogens [4]. Pathogens adapted to their host plants can deliver virulence effector proteins into plant cells, which target key PTI components and inhibit plant defense [5–9]. In turn, plants have evolved resistance (R) proteins to detect directly or indirectly the effector proteins and trigger disease resistance effector-triggered immunity (ETI), which is highly specific and often accompanied by the hypersensitive response (HR) and systemic acquired resistance (SAR). An additional surveillance system for the presence of pathogens is the release or production of endogenous damage associated molecular patterns (DAMPs), including plant cell wall and cutin fragments that are released by the enzymatic action of pathogens and also trigger immune responses [3,10,11]. Thus, plant immunity can be divided in two phases: PTI triggered by PAMPs and ETI triggered by effectors, with the difference being that activated immune responses in ETI are faster and amplified compared to those in PTI [4,12]. ETI and PTI pathways result in activation of an overlapping set of downstream immune responses, suggesting that there is a continuum between PTI and ETI [13]. These downstream defense responses include the activation of multiple signaling pathways and transcription of specific genes that limit pathogen proliferation and/or disease symptom expression. In addition, antimicrobial compounds are produced, reactive oxygen species (ROS) accumulate, cell wall defense mechanisms are activated and defense hormones, such as salicylic acid (SA), ethylene and jasmonic acid (JA) accumulate [4,14–17].

During the last few years, some progress has been made on the defense mechanisms activated in mosses (Bryophytes) during pathogen assault. The moss *Physcomitrella patens* (*P. patens*) is an interesting model plant to perform functional studies of genes involved in stress responses, because its genome has been sequenced, targeted knock-out mutants can be generated by homologous recombination and it has a dominant haploid phase during its life cycle [18–20]. Mosses are non-vascular plants that diverged from flowering plants more than 450 million years ago [21]. *P. patens*, together with the sequenced vascular spikemoss *Selaginella moellendorffii* [22], provide an evolutionary link between green algae and angiosperms, allowing comparative studies of the evolution of plant defense mechanisms and gene function. In nature, mosses are infected with microbial pathogens, resulting in chlorosis and necrosis of plant tissues [23–25]. Necrotrophic pathogens are capable of infecting and colonizing *P. patens* tissues, leading to the activation of defense responses [26–32]. Most likely, *P. patens* utilizes similar mechanisms for pathogen recognition as flowering plants, since chitin (PAMP) [31] and probably cell wall fragments generated by the action of cell wall degrading enzymes from bacterial pathogens (DAMPs) [26] are sensed by *P. patens* cells and typical PRRs and R genes homologues are present in its genome [33–35]. In addition, many of the cellular and molecular defense reactions activated in *P. patens* are similar to those reported in flowering plants. The present paper reviews the current knowledge of defense responses activated in *P. patens* and compares them to those activated in flowering plants after pathogen assault.

## 2. Broad Host Range Pathogens Infect both Mosses and Flowering Plants

Broad host range pathogens are capable of infecting a variety of plant species, including flowering plants and mosses. These are successful pathogens, which have adapted and developed effective invasion strategies causing disease by producing different compounds, including enzymes and toxins that interfere with metabolic targets common to many plant species. In this review, we focus on the broad host range fungus *Botrytis cinerea*, the bacterium *Pectobacterium carotovorum* subsp. *carotovorum* and the oomycetes *Pythium irregulare* and *Pythium debaryanum*. These are necrotrophic pathogens that actively kill host tissue prior to or during colonization and thrive on the contents of dead or dying cells [36].

*B. cinerea* is a necrotrophic fungal pathogen that attacks over 200 different plant species [37] and penetrates plant tissues by producing toxins and multiple cell wall degrading enzymes (CWDEs), including pectinolytic enzymes and cutinases that kill the host cells causing grey mould disease in many crop plants [38]. *B. cinerea* is primarily a pathogen of dicotyledonous plants, but some monocot species, including onions and lilies, are also infected [39,40]. *B. cinerea* also infect *P. patens* plants, producing maceration of the tissues and browning of stems and juvenile protonemal filaments [26,28].

**Figure 1:**
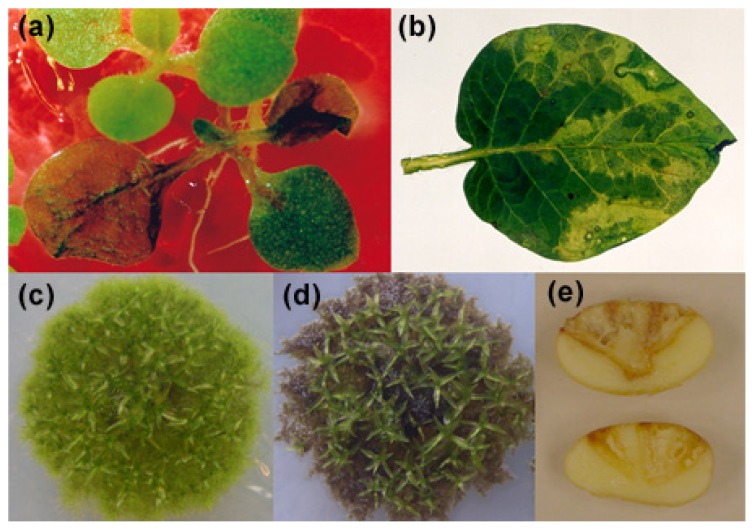
Disease symptoms evidenced by tissue maceration of plants inoculated with *Pectobacterium carotovorum* subsp. *carotovorum* (*P.c. carotovorum*) or treated with elicitors of this pathogen. (**a**) *Nicotiana tabacum* leaves inoculated with *P.c. carotovorum*_SCC3193_ at 48 h post-inoculation; (**b**) *Solanum tuberosum* leaf treated during 72 h with elicitors of *P.c. carotovorum*_SCC3193_; (**c**) water-treated *P. patens* colony; (**d**) *P. patens* colony treated during 48 h with elicitors of *P.c. carotovorum*_SCC1_; (**e**) *Solanum tuberosum* tubers inoculated with *P.c. carotovorum*_SCC3193_ (upper tuber) or treated with elicitors of this strain (lower tuber) during 24 h.

**Figure 2:**
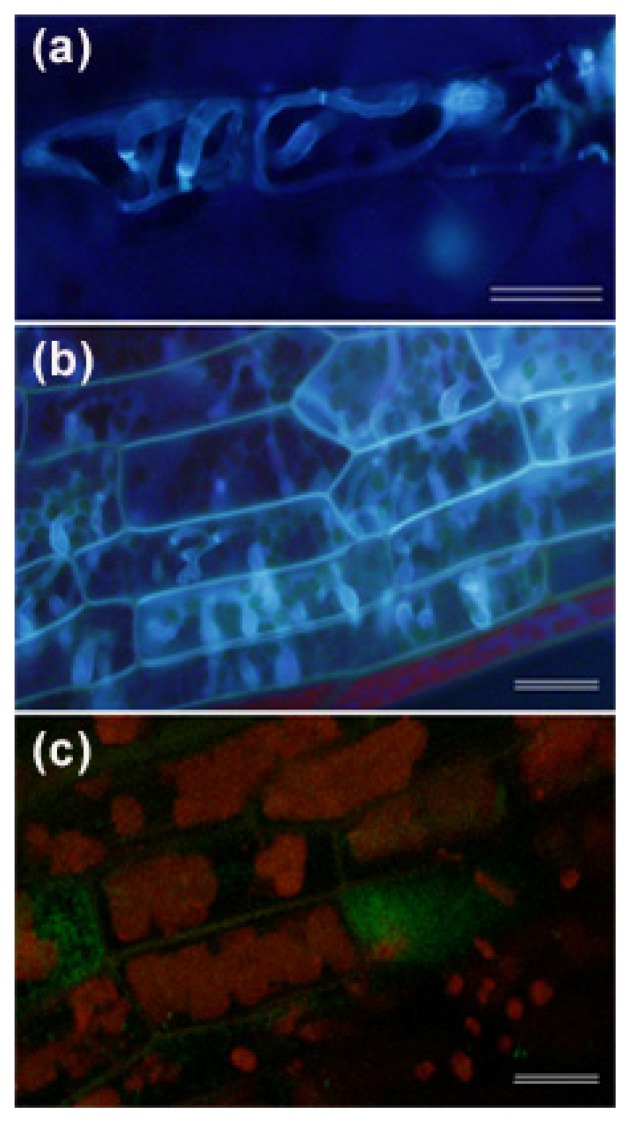
Colonization of *P. patens* leaves by *B. cinerea*, *P. debaryanum* and *P.c. carotovorum*. Stained hyphae are visualized with the fluorescent dye solophenyl flavine 7GFE 500 after 24 h of *B. cinerea* inoculation (**a**) and (**b**) 48 h of *P. debaryanum* inoculation. (**c**) Leaves of *P. patens* inoculated with *P.c. carotovorum*_SCC3193_ carrying a GFP-expressing plasmid at 48 h post-inoculation. The scale bar represents 20 μm.

*P.c. carotovorum* (ex *Erwinia carotovora* subsp. *carotovora*) cause soft rot in a wide range of plant species, including vegetables, potato and Arabidopsis [41]. *P.c. carotovorum* is often described as a brute-force pathogen, because its virulence strategy relies on plant CWDEs, including cellulases, proteases and pectinases, which disrupt host cell integrity and promote tissue maceration [42,43]. Cell-free culture filtrate (CF) containing CWDEs from *P.c. carotovorum* produces similar symptoms (Figure 1) and defense gene expression as those caused by *P.c. carotovorum* infection, demonstrating that CWDEs are the main virulence factors [43–48]. In addition, these CWDEs release cell wall fragments, including oligogalacturonides that act as DAMPS activating an immune response in plant cells evidenced by the activation of defense related genes and phytoalexin accumulation [44,49–51]. Recently, it was shown that two strains of *P.c. carotovorum*, SCC1, harboring the harpin-encoding *hrpN* gene, which is an elicitor of the hypersensitive response (HR) [52], and the HrpN-negative *P.c. carotovorum* strain (SCC3193) [53] infect and cause maceration in leaves of *P. patens* [26]. Green fluorescent protein (GFP) labeled- *P.c. carotovorum*, was detected in the apoplast, as well as the space of *P. patens* invaded leaf cells (Figure 2). Treatments with CFs of these strains also caused symptom development in moss tissues, evidenced by tissue maceration and browning, which was more severe with the HrpN-positive strain, suggesting that harpin may contribute to *P.c. carotovorum* virulence [26].

*Pythium* species are soil-borne vascular pathogens, which infect the plants through the root tissues and under humid conditions cause pre-/post-emergence damping-off and root and stem rots in important crop species. *Pythium* infect host young tissues, and maceration is caused by both toxins and cell wall degrading enzymes, such as pectinases, hemicellulases, cellulases and proteinases [54,55]. *P. irregulare* and *P. debaryanum* infect *P. patens*, producing tissue maceration and browning of young protonemal tissues, stems and leaves [29]. In nature, *Pythium ultimum* infect mosses, causing the formation of areas of dead moss tissues [24]. In all these moss-pathogen interactions, multiple defense reactions are activated in plant cells, although they are not sufficient to stop infection, and after a few days, moss tissues are degraded, leading to plant decay.

## 3. Activation of Cell Wall Associated Defense Responses

**Figure 3:**
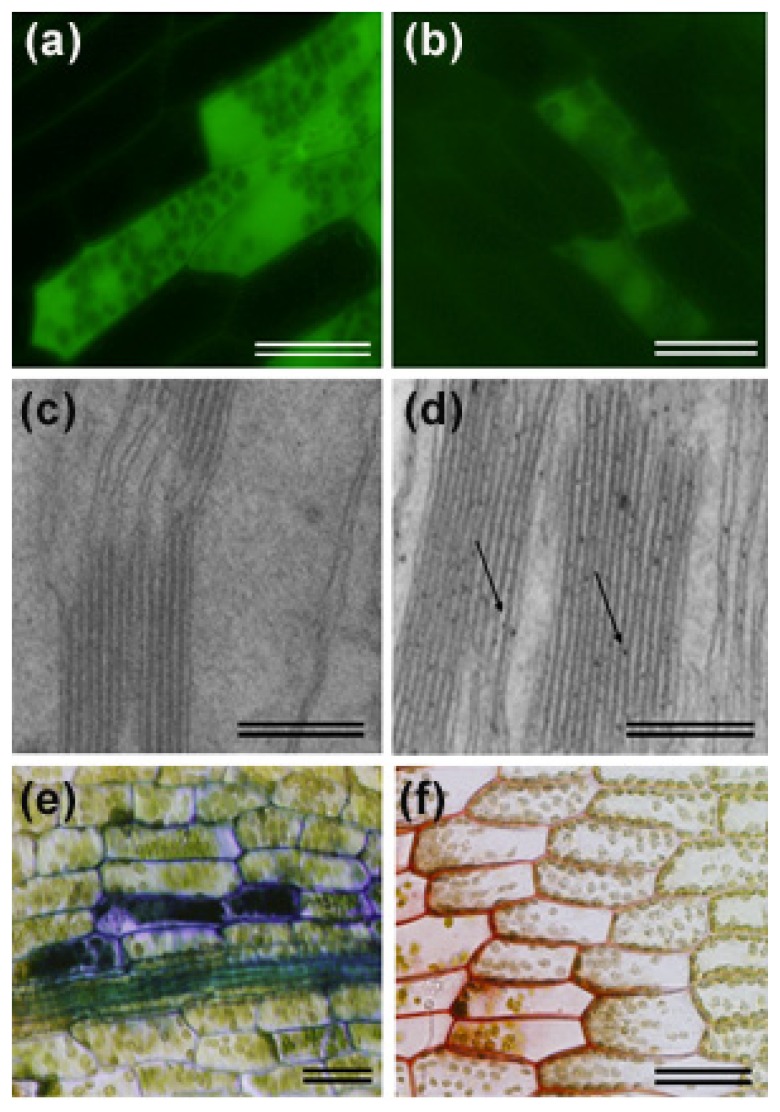
Reactive oxygen species (ROS) production and cell wall reinforcement in pathogen-infected plant tissues. Generation of intracellular ROS was observed using 2′,7′-dichlorodihydrofluorescein diacetate in *P. patens* leaves inoculated with *P. irregulare* (**a**) and *B. cinerea* (**b**) at 24 hpi. Hydrogen peroxide accumulation was detected by cerium chloride staining and transmission electron microscopy in *Solanum tuberosum* leaves treated with water (**c**) and treated with elicitors of *P.c. carotovorum* (**d**). Arrows indicate examples of electron-dense deposits of cerium perhydroxides in chloroplasts. Cell wall associated defenses were detected with toluidine blue staining of a *B. cinerea*-infected leaf (**e**) and safranin-*O* staining of a *P. irregulare* infected leaf (**f**) showing incorporation of phenolic compounds into the cell walls. The scale bar in a, b, e and f represents 20 μm, while in c and d, the scale bar represents 200 nm.

Pathogens are capable of penetrating the plant cell wall and gain access to cellular nutrients. Plant cells have developed pre-invasive structural defenses, including the cuticle and modifications of the cell wall that serve as barriers for the advance of potential pathogens [38,56]. Modification of the plant cell wall is an important defense mechanism operating in the defense response of flowering plants against necrotrophs [57,58]. Reinforcement of the cell wall involves accumulation of phenolic compounds, ROS and callose deposition at attempted penetration sites, making the cell wall less vulnerable to degradation by CWDEs. Callose is a high–molecular weight β-(1,3)-glucan polymer that is usually associated, together with phenolic compounds, polysaccharides and antimicrobial proteins, with cell wall appositions, called papillae, which are proposed to be effective barriers that are induced at the sites of pathogen attack [59,60]. Callose depositions are formed during early stages of pathogen invasion to inhibit pathogen penetration and are sites of accumulation of antimicrobial secondary metabolites [61]. Callose deposition plays a role in the defense response of *Arabidopsis thaliana* against *P. irregulare*, since the callose synthase mutant *pmr4* is more susceptible to this oomycete compared with wild-type plants [62]. Phenolic compounds are also incorporated in cell walls of *Pythium-*infected tissues of flowering plants [63]. Similarly, the *P. patens* defense response against *P. irregulare* and *P. debaryanum* involves the accumulation of phenolic compounds, which were observed in the entire cell wall of infected cells (Figure 3) [29]. In contrast to *P. irregulare*-infected *Arabidopsis* plants [62], callose-containing wall appositions were usually not detected in *Pythium*-infected moss tissues [29]. However, callose depositions were observed when an old *Pythium* inoculum was used and colonization was not extensive, showing that these cell wall appositions can be formed at attempted infection sites, halting the progress of the invading pathogen [29].

Modification of the plant cell wall by the incorporation of phenolic compounds is also an important defense mechanism in the response of flowering plants against *B. cinerea* [57,58]. Increased activity of type III cell wall peroxidases, which probably influence the degree of crosslinking, resulted in enhanced resistance to *B. cinerea* [64]. Upon *B. cinerea* infection, *P. patens* incorporates phenolic compounds in the cell wall and increases expression of dirigent (DIR) encoding gene(s) [28]. DIR proteins are thought to mediate the coupling of monolignol plant phenols to yield lignans and lignins [65], and it is suggested that they participate in the defense response against pathogens [66,67]. Consistently, enzymes involved in monolignol biosynthesis, including putative cinnamoyl-CoA reductases, increase in *Arabidopsis* plants inoculated with *B. cinerea* [68].

The genome of *P. patens* contains orthologs of all the core lignin biosynthetic enzymes with the exception of ferulate 5-hydroxylase (F5H), which converts G (guaiacyl) monolignol to S (syringyl) monolignol [69]. The occurrence of lignins in bryophytes is still controversial, and instead, mosses may have wall-bound phenolics that resemble lignin [70,71]. The lack of genuine lignin together with the absence of S monolignols in *P. patens* could contribute to the high susceptibility observed in *Pythium* and *B. cinerea* infected moss tissues [28,29]. Recently, Lloyd and coworkers suggested that syringyl-type lignols in particular are important for successful defense of flowering plants against *B. cinerea* [72].

## 4. ROS Accumulation and Programmed Cell Death in Pathogen-Infected and Elicitor-Treated Plant Tissues

The production of ROS is one of the earliest plant cell responses following pathogen recognition and is involved in cell wall strengthening via cross-linking of glycoproteins, defense signaling and induction of the hypersensitive response [73]. Plant cells produce ROS after *B. cinerea* attack, which assist fungal colonization, since treatments with antioxidants suppress fungal infection [57]. Aggressiveness of different *B. cinerea* isolates correlates with the amount of H_2_O_2_ and hydroxyl radicals present in leaf tissues during infection [74]. In addition to increased ROS production generated by the host plant as part of a defense mechanism, *B. cinerea* itself produces ROS, including hydrogen peroxide, which accumulates in germinating conidia during the early steps of tissue infection [75,76]. Inactivation of the major *B. cinerea* H_2_O_2_-generating superoxide dismutase (SOD) retarded development of disease lesions, indicating that this enzyme is a virulence factor leading to the accumulation of phytotoxic levels of hydrogen peroxide in plant tissues [77]. Thus, ROS production is an important component of *B. cinerea* virulence, and increased levels of ROS in plant cells contributes to host cell death and favors fungal infection [78]. ROS production also increased in moss tissues after *B. cinerea*, *P. irregulare* and *P. debaryanum* infection (Figure 3) [28,29]. Single cells respond rapidly to *B. cinerea* hyphae contact by generating ROS, suggesting that, like vascular plants [78,79], the oxidative burst is probably induced before and during *B. cinerea* invasion.

*P.c. carotovorum* elicitor treatment also increases ROS production in *P. patens* tissues (Ponce de León *et al.*, unpublished results), similarly to flowering plants [80]. In addition, the fungal elicitor chitin and chitosan caused an oxidative burst in *P. patens* cells [30,32]. The importance of ROS production as a defense mechanism against microbial pathogen in mosses was demonstrated in the *P. patens* class III peroxidase knock-out mutant *Prx34*, which showed enhanced susceptibility to fungal pathogens compared to wild-type *P. patens* plants [30]. This mutant is unable to generate an oxidative burst after elicitor treatment. While a saprophytic fungal isolate of genus *Irpex* and a pathogenic isolate of *Fusarium* sp. caused only mild symptom development in wild-type plants, hyphal growth was abundant and symptoms were severe in *Prx34* knock-out plants, leading to moss decay [30]. Class III peroxidases from flowering plants are known to have antifungal activity [81], and recently, it was shown that the secreted effector Pep1 from the fungus *Ustilago maydis* directly interacts with a class III peroxidase from maize, suppressing the plant defense response by interfering with ROS production [82]. The functional relevance of the Pep1-peroxidase (POX12) interaction was demonstrated with POX12 silenced plants, which were infected by the *pep1* deletion mutant, indicating that inhibition of this peroxidase by Pep1 is crucial for *U. maydis* infection [82]. In addition, PpTSPO1 moss knock-out mutants, which are impaired in mitochondrial protoporphyrin IX uptake and produce elevated levels of intracellular ROS [83], exhibited increased susceptibility to a fungal necrotrophic pathogen, including *Irpex* sp. and *Fusarium avenaceum*, suggesting that PpTSPO1 controls redox homeostasis, which is necessary for efficient resistance against pathogens [32].

Cell death plays a different role in plant response to biotrophs and necrotrophs. The hypersensitive response (HR) is a type of programmed cell death (PCD) with features of two types of cell death recently described, vacuolar cell death and necrotic cell death [84]. HR cell death contributes to resistance to biotrophic pathogens by confining the pathogen and limiting its growth [4]. Biotrophic pathogens actively suppress the HR by using effectors. *Pseudomonas syringae* and *Xanthomonas campestris* deliver 15 to 30 effectors into host cells using type III secretion systems to suppress PTI and ETI, including the HR [85]. In contrast, necrotrophic pathogens actively stimulate the HR, which enhances tissues colonization and host susceptibility. Plant mutants with enhanced cell death have increased resistance to biotrophic pathogens, but higher susceptibility to necrotrophic fungi [86,87]. *B. cinerea* produces nonspecific phytotoxic metabolites, which contribute to cell death on different plant hosts [76]. As part of its invasion strategy, *B. cinerea* promotes PCD in plant cells [78], and studies in flowering plants suggest that *B. cinerea* needs HR to achieve full pathogenicity [78,88]. *Arabidopsis* mutants with an accelerated cell death response are more susceptible to *B. cinerea*, while mutants with reduced or delayed cell death are generally more resistant [89]. *P. patens* also activate an HR-like response after *B. cinerea* colonization, evidenced by protoplast shrinkage, accumulation of ROS and autofluorescent compounds, chloroplasts breakdown and TUNEL (terminal deoxynucleotidyl transferase-mediated dUTP nick end labeling) positive nuclei of infected cells [26,28]. Pathogen-infected *P. patens* tissues also showed other characteristics of PCD, including nucleus condensation and DNA fragmentation, presence of nuclease activities and formation of cytoplasmic vacuoles [31]. Treatments with elicitors, such as CFs of *P.c. carotovorum* and chitosan, also provoked cell death in *P. patens* tissues [26,31]. Harpin proteins from *Pectobacterium* sp. [90,91], *Xanthomonas axonopodis* [92] or *Pseudomonas syringae* [93] elicit HR in flowering plants. Consistently, moss cells treated with the CF of the *P.c. carotovorum* harpin-positive strain SCC1 showed hallmarks of PCD, including protoplast shrinkage, accumulation of autofluorescent compounds and chloroplasts breakdown, while none of these features were detectable in CF treatments with the *P.c. carotovorum* harpin-negative strain SCC3193 [26]. Chitosan induces ROS production and cell death with hallmarks of PCD in young protonemal tissues and gametophores [31]. Interestingly, genes involved in plant PCD, such as those encoding proteases, deoxiribonucleases and ribonucleases and the antiapoptotic Bax Inhibitor-1 (BI-1) are induced after pathogen or elicitor treatment of *P. patens* [31]. The most convincing evidence indicating that genetically programmed cell death occurs in moss cells in response to some pathogens, comes from studies showing that transgenic *P. patens* plants overexpressing BI-1 are more resistance to necrotrophic fungal pathogens [31].

## 5. Induced Expression of Defense-Related Genes and Synthesis of Metabolites

Perception of a pathogen by a plant triggers rapid defense responses via multiple signaling pathways that lead to the induced expression of genes with different roles in defense. These include genes encoding functionally diverse pathogenesis-related (PR) proteins, transcription factors and enzymes involved in the production of metabolites (e.g., phenylpropanoids) and hormones [15,94,95]. Transcriptional reprogramming occurs rapidly after pathogen infection, and in the case of *Arabidopsis*-*B cinerea* interaction, a high-resolution temporal analysis demonstrated that approximately one-third of the *Arabidopsis* genome is differentially expressed during the initial stages of infection [96]. As expected, *P. patens* also sense the presence of pathogens and elicitors and respond rapidly by activating defense gene expression. *B. cinerea*, *P. irregulare* and *P. debaryanum* induce the expression of *PAL* (phenylalanine ammonia-lyase), *CHS* (chalcone synthase) and *LOX1* (lipoxygenase) in *P. patens* tissues [26,28,29]. PAL is a key enzyme in the synthesis of phenylpropanoids, including lignin monomers, phytoalexin antibiotics and the production of SA and CHS is the first enzyme in the synthesis of flavonoids [95]. LOXs are enzymes involved in the synthesis of oxygenated fatty acids (oxylipins), including JA and aldehydes, which play important functions in plant defense against microbial infection and insects [97]. Elicitors of *P.c. carotovorum* also induce *PpPAL*, *PpCHS*, *PpLOX1* and the pathogenesis-related gene *PpPR-1* [26]. ROS-responsive genes encoding alternative oxidase (PpAOX), NADPH-oxidase (PpNOX) and LOX (PpLOX7) are induced by chitosan [32], while *B. cinerea* and *P.c. carotovorum* elicitors induce the expression of *P. patens* genes encoding glutathione S-transferases and ascorbate peroxidases (Ponce de León *et al.*, unpublished data).

Mosses are known to contain a whole range of secondary metabolites, which are not present in flowering plants. The *P. patens* genome has been duplicated 30 and 60 million years ago, and metabolic genes seem to have been retained in excess following duplication, leading probably, in part, to the high versatility of moss metabolism [98]. Some of these metabolites, such as flavonoids, have played important roles in the adaptation of plants to land, to cope with a variety of stresses, including ultraviolet-B (UV-B) radiation, desiccation stress and co-evolving herbivores and pathogens. For example, *P. patens* has a higher number of members composing PAL and CHS multigene families as compared to flowering plants [99,100], and some specific genes could contribute to host defense. Consistently, several genes of the phenylpropanoid pathway leading to flavonoids synthesis, including 4-coumarate:coenzyme A ligase, several CHS and chalcone isomerase are induced in *P. patens* tissues after *P.c. carotovorum* elicitor treatments (Navarrete and Ponce de León *et al.*, unpublished results). Moreover, recent studies showed that *P. patens* accumulated quercetin derivatives in response to UV-B radiation [99]. These flavonoids could also be involved in moss defense responses, since quercetin induces a resistance mechanism in *Arabidopsis* tissues in response to *Pseudomonas syringae* pv. *tomato* DC3000 infection, evidenced by an oxidative burst, callose deposition, and induced expression of *PR-1* and *PAL* [101]. In addition, the *Pythium* and *B. cinerea* inducible PpLOX1 [26,28] can use arachidonic acid as a substrate leading to the production of oxylipins, which are not present in flowering plants [102–104] and could contribute to the *P. patens* defense response. PpLOX1 and PpLOX2 can produce 12-hydroperoxy eicosatetraenoic acid (12-HPETE) from arachidonate, which in turn serves as substrate for a hydroperoxide lyase (HPL) [102,105] or PpLOX1 and PpLOX2, which posses hydroperoxide cleaving activity [102,103], leading to the production of different C8- and C9-oxylipins. *P. patens* HPL can also use 9-hydroperoxides of C18-fatty acids as substrate, producing (2E)-nonenal and C8-volatiles [105]. The aldehyde (2E)-nonenal could contribute to the defense of *P. patens*, since it has antimicrobial activity against certain pathogens, including *Pseudomonas syringae* pv. *tomato* and *Phytophthora infestans* [106].

Chitosan induces the production of secondary metabolites in *P. patens*, such as cyclic diterpenes, and increases transcript levels of genes encoding key biosynthetic enzymes of this metabolic pathway [31,107]. Inducible ent-kaurane–related diterpenoids play important roles in protecting vascular plants against microbial pathogens, as is the case for the causal agent of rice blast disease, *Magnaporthe grisea* [108], and *Rhizopus microsporus* and *Colletotrichum graminicola*, which cause stalk rot in maize [109].

## 6. Defense Hormones

Plant hormones, including SA, JA, ethylene, abscisic acid (ABA) and auxins, are involved in the defense response of flowering plants against pathogens, and the role played by these hormones is related to the particular host-pathogen interaction [110]. In general, SA is effective in mediating plant resistance against biotrophs, whereas JA and ethylene are effective in mediating resistance against necrotrophs [111–114]. The interplay between these defense hormones, both agonistic and antagonistic, will determine the outcome of the interaction and minimizes fitness costs, generating a flexible signaling network that allows fine tuning of the inducible defense mechanisms [110,115,116].

*P. patens* is capable of producing ABA, auxin and cytokinin [117–119], and during the last few years, most studies on moss hormones have been focused on ABA-dependent abiotic stress responses and the regulation of development processes by auxin and cytokinin [120–124]. Until present, only a few studies have been focused on moss hormones in plant-pathogen interactions. The role of ABA in defense responses depends on the infection stage, the type of tissue infected and the specific host pathogen interaction [125]. Evidence indicates that ABA plays a role in the resistance of flowering plants, including stomatal closure, defense gene expression and ROS production/scavenging [57,125–128]. In flowering plants, ABA antagonizes resistance to *B. cinerea*, since ABA-deficient mutants are more resistant to infection [58,62,129]. Consistently, increased ABA levels contribute to the development of grey mould in tomato [57,125]. *B. cinerea*-infected *P. patens* plants showed a small increase in ABA content when mycelium growth was extensive, suggesting that ABA could be produced by *B. cinerea* itself [130] to promote susceptibility by interfering with defense signaling, like the SA pathway, as has been reported previously for flowering plants [131,132].

Bryophytes produce ethylene [133,134] and the *P. patens* genome encodes proteins homologous to ethylene signaling components [18,135]. There are seven putative ethylene receptor proteins in *P. patens* [135] and genes encoding EIN3, EIL and ERF-type components, although the existence of a CTR1 component of ethylene signaling is less clear [136]. A mutation of the presumed ethylene binding site of PpETR7 inhibits the *P. patens* ethylene response, indicating that *P. patens* perceives ethylene using PpETR7 [136]. Ethylene induces defense mechanisms in flowering plants, including the production of phytoalexins, PR proteins, the induction of the phenylpropanoid pathway and cell wall modifications [137]. Resistance against *B. cinerea* is thought to be influenced by ethylene [138–140]. *B. cinerea* produces ethylene itself and can interfere in this way with plant defense signaling [141]. Ethylene production increases in Arabidopsis after *B. cinerea* infection [142], and pretreatment of tomato plants with ethylene results in increased resistance against *B. cinerea*, evidenced by decreased disease symptoms and fungal biomass [137]. In addition, ethylene influenced phenylpropanoid metabolism, leading to accumulation of hydroxycinnamates and monolignols at the plant cell wall, is linked to ethylene-mediated resistance against *B. cinerea* [72]. Although studies on the effect of ethylene on the *P. patens* defense system has not been addressed, the ethylene precursor, 1-aminocyclopropane-1-carboxylic acid (ACC), induces the expression of some defense genes in *P. patens* (Ponce de León *et al.*, unpublished results), suggesting that, like flowering plants, ethylene participates in the moss defense response. The use of the candidate ethylene receptors mutant *Ppetr7-1* will contribute to understanding the role played by ethylene in the defense of *P. patens* against pathogen infection.

Until very recently, it was unknown if bryophytes produce SA and JA. The *P. patens* genome has 14 putative genes encoding PALs [99] and several putative homologues of isochorismate synthases, supporting the synthesis of SA in this moss. In addition, *P. patens* synthesizes at least seven LOXs [104], two allene oxide synthase (AOS) [143,144], three allene oxide cyclase (AOC) [145,146] and several putative 12-oxo-phytodienoic acid (OPDA) reductases genes [147,148], which encodes enzymes leading to the production of JA. Until present, enzymatic activity has been confirmed for LOXs, AOSs and AOC [104,143–146], although OPR3 activity, which is the only enzyme capable of converting cis-(+)-OPDA to JA, is still missing [147]. Like flowering plants, *P. patens* responds to *B. cinerea* and *P. irregulare* infection by increasing endogenous levels of the precursor of JA, OPDA [28,29,62,149]. Transcript levels of genes encoding enzymes involved in OPDA biosynthesis, including LOX and AOS, are induced in *B. cinerea* infected tissues [28]. OPDA reductase transcript levels also increase in *P. patens* tissues in response to *B. cinerea* inoculation [28]. However, JA could not be detected in healthy, pathogen-infected, elicitor-treated or wounded *P. patens* tissues, suggesting that oxylipins are not further metabolized to JA [28,145,150]. Thus, cis-(+)-OPDA might function as a signaling molecule in *P. patens* instead of JA. Studies with the *Arabidopsis opr3* mutant have shown that OPDA is active as a defense signal against pathogens and regulates defense gene expression [150–152]. Interestingly, moss tissues respond to the presence of OPDA and JA by decreasing rhizoid length and moss colony size [28], similarly to the reduced growth of seedlings and roots observed in OPDA and Methyl Jasmonate (MeJA) treated *Arabidopsis* [153–156]. Moreover, JA, MeJA and OPDA induced the expression of *PAL* in *P. patens*, showing that the presence of these oxylipins is sensed by this moss and signal transduction events are activated, leading to increased levels of defense-related transcripts [29]. The *P. patens* genome has six putative genes encoding the JA-isoleucine receptor COI (coronatine insensitive) and six encoding the repressor JAZ (jasmonate ZIM-domain) [157]. *P. patens* COI-like receptors could bind other oxylipins instead of JA-isoleucine, including cis-(+)-OPDA and/or cis-(+)-OPDA-isoleucine. Thus, the JA signaling pathway could have evolved after divergence of bryophytes and vascular plants. In addition, the similarities between the auxin receptor (TIR1) and COI1 suggest that COI-1 could have evolved from a TIR1 ancestor by gene duplication, leading to perception of JA-isoleucine by successive mutations [157].

Salicylic acid levels increase in response to *B. cinerea* infection in flowering plants [158,159] and in *P. patens* [28]. Like flowering plants, SA seems to play an important role in the defense of *P. patens* against microbial pathogens. SA treatment of moss tissues induces the expression of the defense gene *PAL* [28], and SA application induced defense mechanisms and increased resistance to *P.c. carotovorum* in *P. patens* colonies [160]. SA-mediated resistance could be due to activation of similar defense mechanisms in mosses and flowering plants, since exogenous SA application to tobacco plants also increase resistance against *P.c. carotovorum* [161]. In flowering plants, SA plays a key role in the activation of defense mechanisms associated with the HR and participates in a feedback amplification loop, both upstream and downstream of cell death [162,163]. The generation of SA-deficient NahG transgenic moss plants will help to elucidate SA involvement in moss defense, including the HR-like response.

## 7. Conclusions

During land colonization, plants gradually evolved defense strategies to cope with radiation, desiccation stress and airborne pathogens by newly acquired specialized metabolic pathways, such as the phenylpropanoid metabolism. Recently, significant progress has been made on sequencing genomes of plants that occupy interesting positions within the evolutionary history of plants, including the non-vascular moss *P. patens* and the vascular spikemoss *S. moellendorffii* [18,22]. *P. patens* occupies a key position halfway between green algae and flowering plants, allowing evolutionary and comparative studies of defense mechanisms across the green plant lineage. Interestingly, it was recently shown that *P. patens* has acquired genes related directly or indirectly with defense mechanisms by means of horizontal gene transfer from fungi and viruses [164]. The possible uptake of foreign DNA from fungi associated with early land plants could have facilitated the transition to a hostile land environment [164,165]. *P. patens* respond to pathogen infection or elicitor treatment by inducing defense-related gene expression and producing metabolites and hormones that could play different roles in defense. Several defense mechanisms are shared between *P. patens* and flowering plants, and functional conservation of some signaling pathways probably indicate common ancestral defense strategies [28–30,32,136]. While the JA signaling pathway may have evolved after the divergence of bryophytes and vascular plants, ethylene, ABA and SA likely have their origins in the early stages of land colonization. The use of *P. patens* mutants in key components of these signaling pathways will help to determine the role played by these hormones in moss defense. *P. patens* also offers the possibility to identify novel metabolites, some of which are not present in flowering plants, including arachidonic acid-derived oxylipins that could play a role in defense responses. In addition, experimentation with *P. patens* could help to unravel defense pathways and gene functions in plants through the generation of knock-out mutants and single point mutations of genes involved in disease resistance and to identify clear mutant phenotypes due to the presence of a dominant gametophytic haploid phase [19]. Large-scale analyses of transcripts from pathogen-infected or elicitor-treated moss plants together with functional genomic and comparative studies with flowering plants will help to identify key components in the plant defense response and to design strategies to enhance plant resistance to biotic stress.
